# Volatile Fingerprinting and Regional Differentiation of Safflower (*Carthamus tinctorius* L.) Using GC–IMS Combined with OPLS-DA

**DOI:** 10.3390/foods14193381

**Published:** 2025-09-29

**Authors:** Jiaqi Liu, Hao Duan, Li Wang, Rui Qin, Jiao Liu, Hong Liu, Shuyuan Bao, Wenjie Yan

**Affiliations:** 1 College of Biochemical Engineering, Beijing Union University, Beijing 100023, China; liujiaqi0711@foxmail.com (J.L.); dhuanao@163.com (H.D.); 2 Metrology and Food & Drug Testing Center of Shunyi, Beijing 100029, China; wanglisysyj@sina.com; 3 School of Life Sciences, South-Central Minzu University, Wuhan 430074, China; qinrui@scuec.edu.cn (R.Q.); jiao.liu@scuec.edu.cn (J.L.); liuhong@scuec.edu.cn (H.L.); 4 Xiangya School of Public Health, Central South University, Changsha 410083, China; 8304230626@csu.edu.cn

**Keywords:** safflower, volatile organic compounds, geographical origin, GC–IMS, OPLS–DA, hierarchical clustering

## Abstract

This study aimed to systematically characterize the volatile organic compound (VOC) profiles of safflower (*Carthamus tinctorius* L.) from eight major production regions, providing a scientific basis for quality evaluation and geographical traceability. VOC profiling was conducted using gas chromatography–ion mobility spectrometry (GC–IMS), and regional differences were assessed through multivariate statistical analyses, including Principal Component Analysis (PCA), Orthogonal Partial Least Squares Discriminant Analysis (OPLS–DA), Euclidean distance, and hierarchical clustering. Key differential compounds were identified by variable importance in projection (VIP) and relative odor activity value (ROAV) analyses, with aldehydes and esters emerging as the primary contributors to the discrimination of samples across regions. VOC fingerprints of safflower were further established, and a combined VIP–ROAV strategy was proposed for the screening of characteristic compounds. These findings provide a reliable reference for safflower quality control and offer practical guidance for its geographical authentication in the food industry.

## 1. Introduction

Safflower (*Carthamus tinctorius* L.) is an annual herb of the family Asteraceae that has long been cultivated in China, India, Iran, and other countries, serving both medicinal and dietary purposes [1,2]. Modern pharmacological studies have demonstrated that safflower exhibits diverse biological activities, including antioxidant [3], anti-inflammatory [4], antiplatelet aggregation [5], antitumor [6], immunomodulatory [7], as well as microcirculation-improving and lipid- and glucose-regulating effects [8]. Consequently, it has been widely applied in traditional Chinese medicine, functional foods, and the development of natural flavoring agents [9,10].

Beyond its pharmacological functions, the aroma of safflower is a critical sensory attribute influencing quality and consumer acceptance. This aroma mainly arises from a wide array of volatile organic compounds (VOCs), such as acids, aldehydes, ketones, esters, alcohols, and furans [11,12,13,14]. These compounds impart distinctive floral, fruity, fresh, and fatty notes and significantly affect the sensory quality and market value of food products. From a food science perspective, a stable and interpretable aroma fingerprint not only underpins raw material grading and geographical indication protection but also plays a vital role in process monitoring, shelf-life assessment, and quality consistency [12,15,16].

With the increasing demand for authenticity verification and flavor stability, chemometric approaches based on VOC profiling have become important tools for ensuring food quality and safety [16]. In crops such as coffee, tea, and saffron, VOC-based chemical fingerprints have proven highly effective for geographical origin tracing and authenticity verification. For instance, integrating Headspace–Gas Chromatography–Ion Mobility Spectrometry (HS-GC-IMS) or Headspace Solid-Phase Microextraction–Gas Chromatography–Mass Spectrometry (HS-SPME-GC-MS) with PCA, OPLS-DA, or deep learning models has enabled the discrimination of saffron origin and authenticity with accuracies above 90% [17,18]. Similar methods have yielded promising results in the origin discrimination and flavor consistency evaluation of coffee and tea [15,19], underscoring the broad potential of combining chemometrics with volatile analysis for food traceability, quality control, and market supervision [15,20,21].

Recently, GC–IMS has been widely employed for aroma analysis of foods and natural products due to its high sensitivity, rapid detection, simple preparation, high throughput, and low cost [22,23]. This technique generates intuitive fingerprint spectra and, when combined with multivariate statistical methods such as PCA, OPLS–DA, and cluster analysis [24], effectively identifies region-specific compounds in a multidimensional chemical space. It has shown considerable potential for geographical origin tracing and quality control in foods such as meat, oils, and dairy products [18,25,26]. However, systematic comparative studies across multiple production regions and comprehensive analyses of safflower aroma characteristics remain scarce.

Therefore, this study aims to characterize the VOC composition of safflower from eight major production regions using GC–IMS, and to evaluate regional aroma differences through PCA, OPLS–DA, Euclidean distance, and hierarchical clustering, with the objective of identifying key differential markers. By establishing VOC fingerprints, this study provides a scientific basis for safflower traceability and quality evaluation while offering data to support its application in food quality control and functional product development. Therefore, this study aimed to systematically characterize VOCs of safflower from eight major producing regions in China using GC–IMS. Regional differences in aroma profiles were further evaluated through PCA, OPLS–DA, Euclidean distance, and hierarchical clustering, with the objective of identifying key discriminant markers. Notably, although GC–IMS provides advantages in sensitivity and rapid detection, it still has limitations in the unambiguous identification of unknown peaks. Future studies should therefore combine GC–MS with authentic standards and sensory evaluation to enhance reliability. By establishing VOC fingerprint profiles of safflower, this study provides a scientific basis for origin authentication and quality evaluation. Similar research has also been reported in saffron (*Crocus sativus* L.) [12,15,27], which complements the present findings and underscores the broad applicability and value of GC–IMS in elucidating aroma characteristics and tracing the geographical origin of medicinal and edible plants.

## 2. Materials and Methods

### 2.1. Sample Information

**Figure 1:**
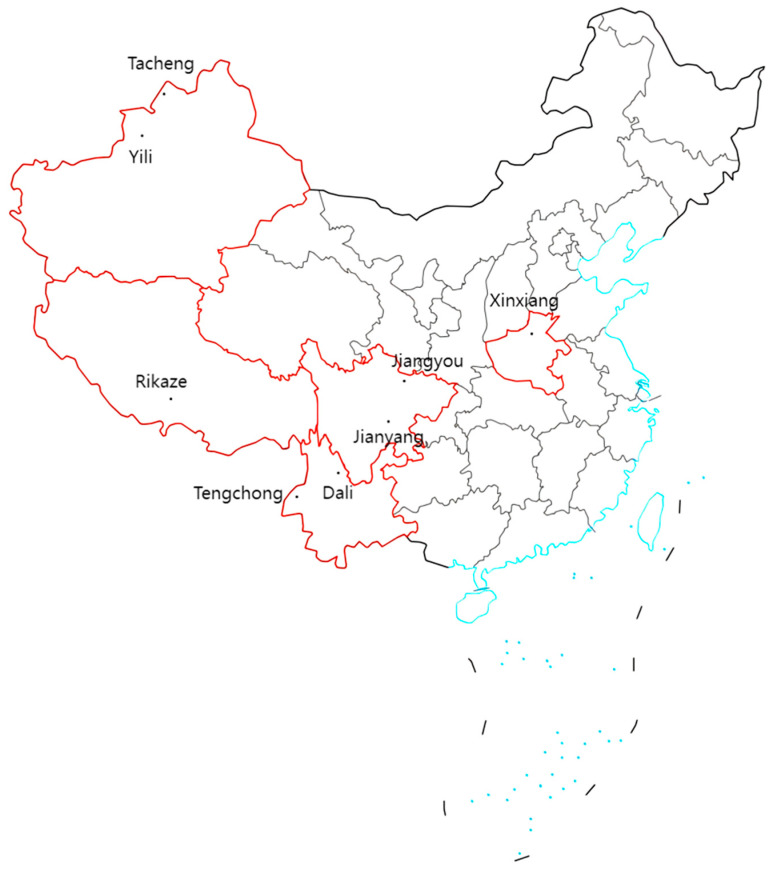
Geographical distribution of safflower (*Carthamus tinctorius* L.) sampling sites across eight major production regions in China (map source: Standard Map Service, Ministry of Natural Resources of China). Red lines indicate the sampled provinces, blue lines represent the coastline, and dashed boxes denote national boundaries.

**Figure 2:**
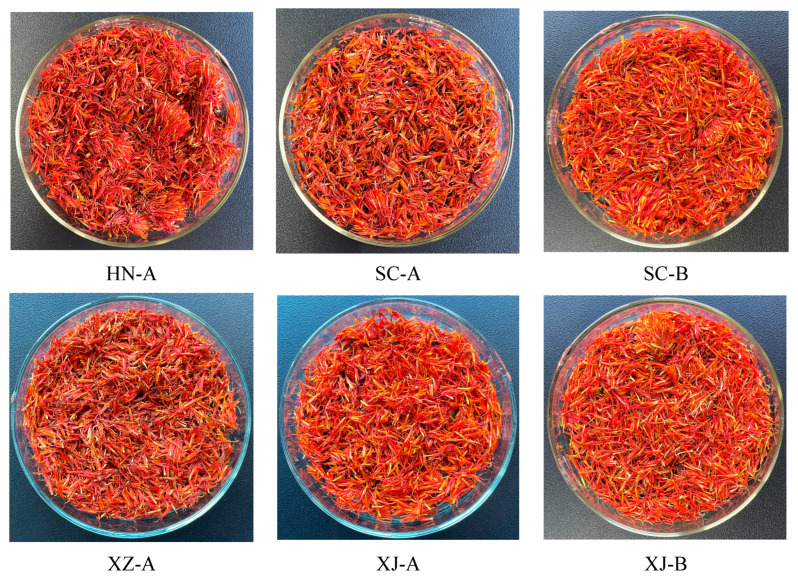

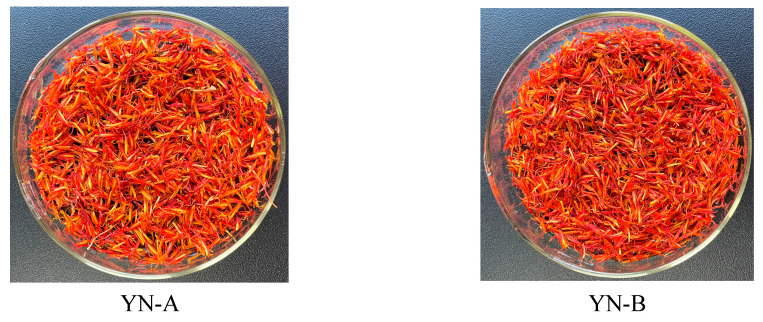
Representative safflower (*Carthamus tinctorius* L.) samples from eight producing regions in China. Sample codes: YN-A (Tengchong, Yunnan), YN-B (Dali, Yunnan), SC-A (Jianyang, Sichuan), SC-B (Jiangyou, Sichuan), XJ-A (Yili, Xinjiang), XJ-B (Tacheng, Xinjiang), HN-A (Xinxiang, Henan), and XZ-A (Rikaze, Tibet).

Safflower (*Carthamus tinctorius* L.) samples were collected from eight major producing regions in China: Tengchong and Dali (Yunnan), Jianyang and Jiangyou (Sichuan), Yili and Tacheng (Xinjiang), Rikaze (Tibet), and Xinxiang (Henan). The geographical distribution of these sampling sites is shown in Figure 1, which illustrates the ecological diversity across regions. At harvest, flowers with intact morphology and vivid coloration were selected, rinsed with distilled water, air-dried in a ventilated shaded environment, and stored in airtight bags until a constant weight was reached. The samples were designated as YN-A (Tengchong), YN-B (Dali), SC-A (Jianyang), SC-B (Jiangyou), XJ-A (Yili), XJ-B (Tacheng), HN-A (Xinxiang), and XZ-A (Rikaze). The regional samples are shown in Figure 2.

Each sampling site represented distinct environmental conditions, as shown in Table 1, Xinxiang in Henan had hot, humid summers and cold winters typical of the North China Plain. Jianyang in Sichuan, within the basin interior, was warm and rainy, whereas Jiangyou on the basin margin was slightly cooler with greater topographic variation. Dali in Yunnan, located in a plateau basin, experienced high elevation, strong solar radiation, and relatively cool conditions, while Tengchong in Yunnan, in mountainous terrain, received heavy monsoon rainfall and maintained mild temperatures. Yili in Xinjiang, protected by the Tianshan Mountains, formed a comparatively moist river valley oasis, in sharp contrast to Tacheng, which was extremely arid with large diurnal temperature fluctuations. Rikaze in Tibet represented the most extreme site, with very high elevation, low annual temperatures, strong solar radiation, and scarce rainfall. Together, these sites spanned plains, basins, plateaus, valleys, arid basins, and alpine zones, providing sharp gradients in climate and geography that are expected to influence safflower growth and volatile compound accumulation.

### 2.2. Instruments and Reagents

#### 2.2.1. Reagents

Analytical grade n-ketones, including 2-butanone, 2-pentanone, 2-hexanone, 2-heptanone, 2-octanone, and 2-nonanone (C4–C9), were purchased from Aladdin (Shanghai, China). These compounds were used as external references for RI calibration, providing a reference range of approximately 700–1200 on the MXT-WAX column. High-purity nitrogen gas (99.999%) was used. Headspace vials (20 mL) were obtained from (Shandong Hanon Scientific Instrument Co., Ltd., Jinan, China). An MXT-WAX capillary column (15 m × 0.53 mm, 1.0 μm) was supplied by Restek (Bellefonte, PA, USA).

#### 2.2.2. Instruments

A gas chromatography–ion mobility spectrometer (FlavourSpec^®^, G.A.S., Dortmund, Germany) equipped with a static headspace autosampler (CTC-PAL 3, CTC Analytics AG, Zwingen, Switzerland) was used. Data were processed with VOCal software (v0.4.10, G.A.S., Dortmund, Germany).

### 2.3. Analysis of Volatile Organic Compounds by GC–IMS

#### 2.3.1. Sample Preparation and Injection Conditions

The stored safflower samples were carefully selected by removing broken, moldy, or discolored petals, while retaining intact dried flowers with uniform bright red or dark red coloration. The samples were ground and sieved through an 80-mesh screen. Precisely 0.50 g of safflower powder (±0.001 g) was weighed, transferred into a 20 mL headspace vial, and sealed. The vial was incubated in a water bath at 80 °C for 15 min before direct injection. Each sample was tested three times to guarantee reliability and consistency.

#### 2.3.2. GC Conditions

The injector was maintained at 250 °C and the column at 60 °C. Nitrogen (≥99.999%) served as the carrier gas. The flow program began at 2.0 mL/min for 2 min, then rose to 10.0 mL/min within 8 min, increased further to 100.0 mL/min over the next 10 min, and finally reached 150.0 mL/min within another 10 min, which was then held constant for 15 min. The total run time was 45 min, and the injection port temperature was maintained at 80 °C [13].

#### 2.3.3. IMS Conditions

A tritium (^3^H) ionization source was used. The drift tube length was 53 mm with an electric field strength of 500 V/cm, and the drift tube temperature was maintained at 45 °C. High-purity nitrogen (≥99.999%) was used as the drift gas at a flow rate of 75.0 mL/min. Analyses were performed in positive ion mode.

### 2.4. Calculation of Relative Odor Activity Values (ROAVs)

The relative odor activity value (ROAV) method was applied to quantify the contribution of each volatile compound to the overall aroma profile [28]. The ROAV of the compound exerting the greatest impact on aroma (ROAV_max_) was defined as 100 [29], and the ROAV of all other compounds (ROAV_i_) was calculated relative to this reference. The calculation is expressed as follows:

$${ROAV}_{i}\approx \frac{C_{i}}{T_{i}}\times \frac{T_{max}}{C_{max}}\times 100$$

where C_i_ and T_i_ represent the relative content and odor threshold of compound i, respectively, while C_max_ and T_max_ correspond to the relative content and threshold of the compound contributing most to the aroma [30]. Compounds with ROAV > 1 are viewed as the principal contributors to aroma, while those with values between 0.1 and 1 function as modifiers that adjust the overall flavor profile.

### 2.5. Data Processing and Statistical Analysis

A calibration curve for retention time and retention index (RI) was generated with a mixed standard solution containing six ketones. The retention index (RI) of each analyte was calculated from its retention time, and compound identification was performed by matching with the GC retention index database (NIST 2020) and the IMS drift time library within VOCal software. The VOCal software (Reporter, Gallery Plot, and Dynamic PCA modules) was used to generate three-dimensional spectra, two-dimensional spectra, differential spectra, fingerprint plots, and PCA score plots of volatile compounds for comparison among samples. OPLS-DA plots were generated using the online platform Metware Cloud (https://cloud.metware.cn, accessed on 13 August 2025). In addition, hierarchical clustering heatmaps were created using the OmicStudio platform (https://www.omicstudio.cn/tool, accessed on 12 August 2025) to visually compare and analyze differences in volatile compounds among samples. A one-way ANOVA was conducted, followed by Duncan’s post hoc test, and results with *p* < 0.05 were regarded as statistically significant.

## 3. Results and Discussion

### 3.1. Overall GC–IMS Spectra and Preliminary Differences

GC–IMS was applied to profile the volatile organic compounds (VOCs) in safflower from different regions and to provide an integrated view of their aroma characteristics. This technique enables both qualitative and semi-quantitative assessment of volatile constituents, providing an effective approach to compare chemical differences among samples.

**Figure 3:**
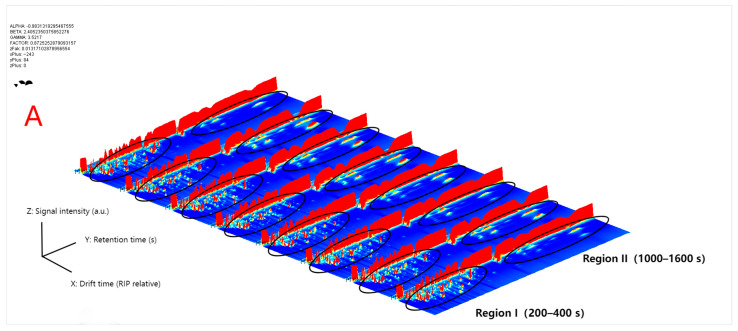

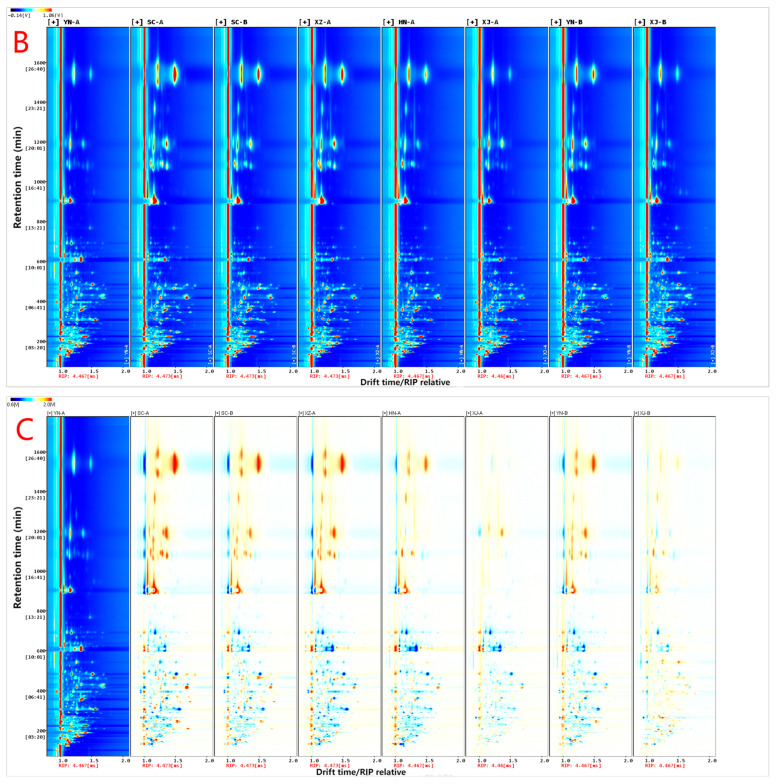
Overall GC–IMS spectra and difference plots. (**A**) Three-dimensional topographic map: *X*-axis represents drift time (relative to RIP), *Y*-axis represents retention time (s), and *Z*-axis represents signal intensity. (**B**) Two-dimensional spectrum: color scale from blue to red indicates increasing relative intensity. Signal intensity (a.u.), Blue = Low, Red = High. (**C**) Difference spectrum with YN-A as the reference: red indicates an increase, and blue indicates a decrease. All samples were analyzed in triplicate (n = 3). Signal intensity (a.u.), Blue = Low, Red = High. Circles indicate the identified volatile organic compounds (VOCs), “+” symbols mark the reference positions, and the color intensity (red to blue) corresponds to the signal strength.

To comprehensively characterize the volatile organic compounds (VOCs) of safflower from different regions, GC–IMS was employed to generate three-dimensional spectra, two-dimensional fingerprint maps, and differential plots (Figure 3A–C). As shown in Figure 3A, the three-dimensional chromatogram provides an intuitive visualization of retention time (*Y*-axis), drift time (*X*-axis, relative to RIP), and signal intensity (*Z*-axis). Each dot corresponds to an analytical signal (AS), with the color scale ranging from blue to red to indicate increasing signal intensity [31]. Overall, two major signal clusters were observed across the samples: Region I (200–400 s), mainly corresponding to low-molecular-weight and highly volatile compounds, and Region II (1000–1600 s), associated with medium- to high-boiling-point compounds. Although the overall distribution patterns appeared similar, marked differences were evident in signal intensities among regions.

The two-dimensional fingerprint plots (Figure 3B) further illustrated the characteristic “chemical fingerprints” of safflower samples from different regions. In these plots, the vertical axis corresponds to retention time, the horizontal axis to drift time, and the color gradient from blue to red reflects the increase in signal intensity [21]. The results showed that peaks in Region I appeared as continuous bands, whereas those in Region II formed island-like hotspots. For example, XJ-A and XJ-B exhibited markedly stronger signals in Region I, while SC-A displayed more concentrated clusters of high-intensity red peaks in Region II. In contrast, XZ-A presented generally weaker signals, whereas YN-B and HN-A shared similar peak patterns.

On this basis, a difference spectrum using YN-A as the reference (Figure 3C) further highlighted regional variations. Red areas indicate compounds with stronger signals relative to the reference, whereas blue areas represent reduced signals. The results showed that SC-A exhibited pronounced red signals around 2200–2400 s and approximately 1.2 RIP, which were likely associated with the enrichment of esters and aldehydes. XJ-B displayed generally enhanced responses in Region I, along with more scattered red signals at higher retention times. In contrast, XZ-A showed extensive blue regions, suggesting an overall lower abundance of volatile compounds.

In summary, the GC–IMS spectra, presented in three-dimensional, two-dimensional, and differential formats, not only revealed the common distribution patterns of safflower samples but also highlighted regional differences in characteristic peak intensities and compound distributions. These preliminary findings provide a solid foundation for subsequent qualitative and quantitative analyses, as well as multivariate statistical discrimination such as PCA and OPLS-DA.

### 3.2. Fingerprint Profiles and Similarity Quantification

**Figure 4:**
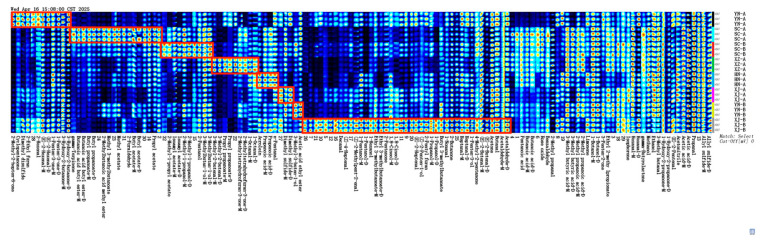
GC–IMS volatile fingerprint maps of safflower samples. Each row denotes a sample and each column corresponds to the same VOC across samples. The color scale indicates relative peak levels, allowing straightforward comparison of global variation and reproducibility among replicates. Red frames highlight the regions corresponding to differential volatile compounds among safflower samples.

Based on the peak table information from Figure 3, as shown in Figure 4 a “sample × peak” fingerprint matrix was constructed, and Euclidean distance (ED) was used to quantify the differences among samples (Table 2). The heatmap results showed that all eight samples exhibited distinct individual characteristics in their overall VOC distributions. XJ-A displayed relatively high signal intensities, particularly enriched in esters and medium-chain aldehydes, reflecting a complex and intense aroma profile. XJ-B presented a greater number of differential peaks, being enriched not only in esters and aldehydes but also in alcohols and furans, further enhancing its discriminability. YN-A was characterized by oxygenated ketones (e.g., 6-methyl-5-hepten-2-one) and sulfur-containing compounds (e.g., dimethyl disulfide), conferring a pungent and spicy flavor. In contrast, YN-B was mainly enriched in alcohols and esters, such as 3-methyl-3-buten-1-ol and ethyl acetate, although its overall abundance was relatively low. SC-A showed a marked enhancement in the ester region (e.g., butyl butyrate and propyl acetate), exhibiting typical fruity characteristics. SC-B presented a more balanced signal distribution but displayed stronger responses in the aldehyde region, particularly hexanal and nonanal. XZ-A contained higher levels of compounds such as 3-methyl-2-butenal and octanal, suggesting a more complex volatile composition, whereas HN-A was dominated by low-molecular-weight aldehydes (e.g., acrolein and pentanal) with an overall weaker signal intensity.

These differential patterns were highly consistent with the difference spectra (Figure 3C) and echoed findings from GC–IMS analyses of other medicinal and edible plants such as ginseng and turmeric [32,33], indicating that the chemical differences observed in safflower not only reflect regional characteristics but are also closely associated with the regulation of secondary metabolism by environmental and geographical factors.

### 3.3. Identification and Classification of Volatile Compounds

In this study, a total of 140 volatile features were detected by GC–IMS, including 108 putatively identified VOCs and 32 unknown compounds (see Supplementary Table S1). Among them, aldehydes (28), esters (22), and alcohols (18) were the predominant classes, followed by ketones (15), acids (10), and furans (15). The top 15 compounds ranked by average relative abundance are listed in Table 3, with hexanal, ethyl acetate, nonanal, and 3-methyl-1-butanol being the most abundant, thereby forming the core framework of safflower volatiles. This result is consistent with previous findings in medicinal and edible plants such as tea and wine, underscoring the universal importance of aldehydes and esters in the composition of plant volatiles.

**Figure 5:**
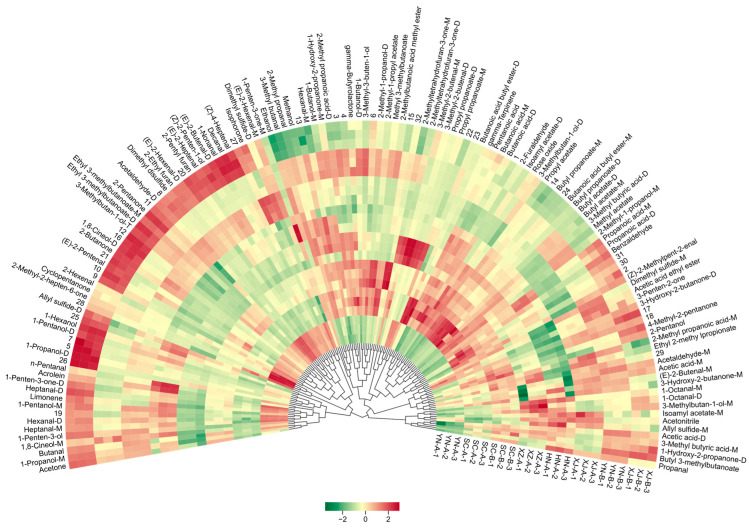
Full-spectrum heatmap combined with hierarchical cluster analysis (HCA) based on all identified VOCs. The central dendrogram illustrates sample clustering using Euclidean distance and Ward’s linkage, while the outer concentric rings represent 108 VOCs. The color gradient (green–beige–red) reflects relative compound abundance across samples, derived from log_1_p-transformed and z-score–normalized peak intensities (green = low, beige = medium, red = high). Unidentified compounds were assigned numeric codes (e.g., 1, 2, …) for reference.

The circular fan-shaped heatmap constructed from the 108 identified VOCs (Figure 5) further revealed differences among the samples. The central clustering results showed that XJ-B was the most distinct from the other samples, occupying a clearly separate position and exhibiting unique chemical characteristics. In contrast, HN-A and XZ-A displayed overall weaker signals and were positioned at a certain distance from the remaining samples. In terms of specific compound distribution, SC-A showed a pronounced enhancement in the ester region, being particularly enriched in propyl acetate, isoamyl acetate, and butyl butyrate, which imparted strong fruity characteristics. YN-A was characterized by high levels of dimethyl disulfide and 6-methyl-5-hepten-2-one, contributing to a pungent aroma. In contrast, XJ-B was markedly enriched in medium-chain aldehydes (e.g., hexanal and nonanal) and alcohols (e.g., 3-methyl-1-butanol and isobutanol), resulting in a more complex aroma profile [34,35]. These differential compounds not only accounted for the major sources of variation among samples but also served as potential markers for regional discrimination.

This pattern was highly consistent with the fingerprinting and Euclidean distance matrix analyses, further indicating that environmental and geographical factors play a significant role in regulating the secondary metabolite composition of safflower. Moreover, comparison with previous studies revealed that aldehydes (e.g., hexanal and nonanal) and esters (e.g., propyl acetate and isoamyl acetate) are likewise important discriminant compounds in other medicinal and edible plants, including chrysanthemum and goji berry. In contrast to saffron (*Crocus sativus* L), where furan derivatives represent a higher proportion [12], furan compounds in safflower (e.g., tetrahydrofuranone) were generally less abundant, thereby highlighting the unique aroma profile of safflower. The circular heatmap provided a straightforward visualization of these differences, with particularly pronounced separations observed for SC-A, YN-A, and XJ-B. Collectively, these findings not only confirm the chemical diversity of safflower across regions but also underscore the potential of GC–IMS as a robust tool for origin authentication and aroma characterization.

These results indicate that the distribution of compound classes was generally consistent among safflower samples from different regions, while the main differences were reflected in the abundance and composition of specific compounds. Such variations will be further clarified in the subsequent heatmap and multivariate analyses.

**Figure 6:**
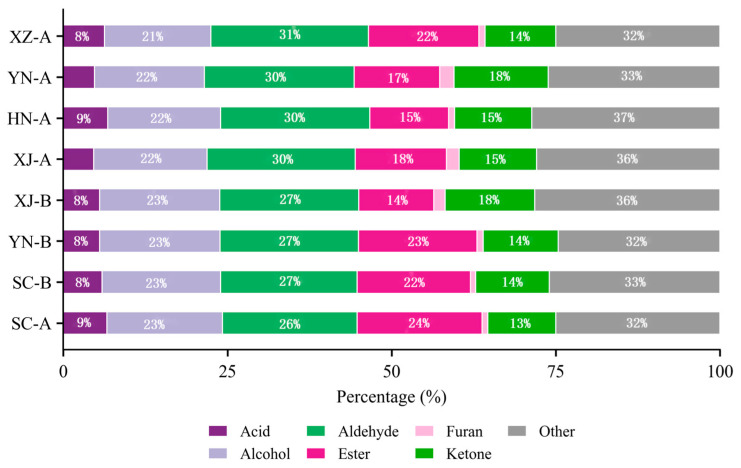
Relative distribution of volatile organic compound (VOC) classes in safflower samples from eight producing regions.

At the chemical class level, as shown in Figure 6 aldehydes (26–31%), esters (15–24%), and alcohols (21–23%) were the predominant constituents across all eight samples. The overall proportions showed little variation, indicating considerable stability in the major metabolite categories of safflower. Nevertheless, some differences were still evident. For example, SC-A exhibited the highest proportion of esters (24%), suggesting a pronounced fruity potential; HN-A contained slightly higher levels of acids (9%) than the other samples; XJ-B was relatively enriched in ketones (18%); whereas YN-B, despite having a relatively high ester proportion (23%), displayed an overall lower VOC abundance.

These results indicate that the distribution of compound classes was generally consistent among safflower samples from different regions, while the main differences were reflected in the abundance and composition of specific compounds. Such variations will be further clarified in the subsequent heatmap and multivariate analyses.

### 3.4. Multivariate Statistical Separation and Model Validation

#### 3.4.1. PCA

The integration of GC–IMS with PCA enables clear visualization of chemical variations and facilitates the identification of major components and their interrelationships [36]. As an unsupervised multivariate statistical tool [37,38], PCA can capture both common patterns and differences among samples [39] and has been widely applied in cultivar classification studies [40]. By reducing dimensionality, PCA simplifies the original variables while preserving the essential information [41,42]. In this study, PCA was applied to differentiate the aroma profiles of safflower samples. Samples with similar aroma characteristics tended to overlap or cluster closely in the score plots, whereas those with distinct differences were clearly separated [43].

**Figure 7:**
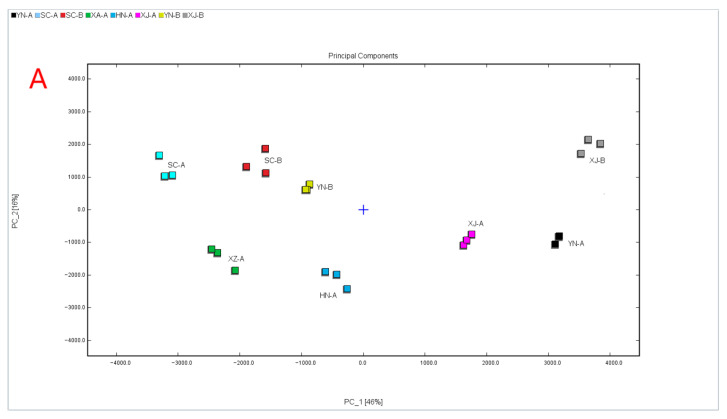

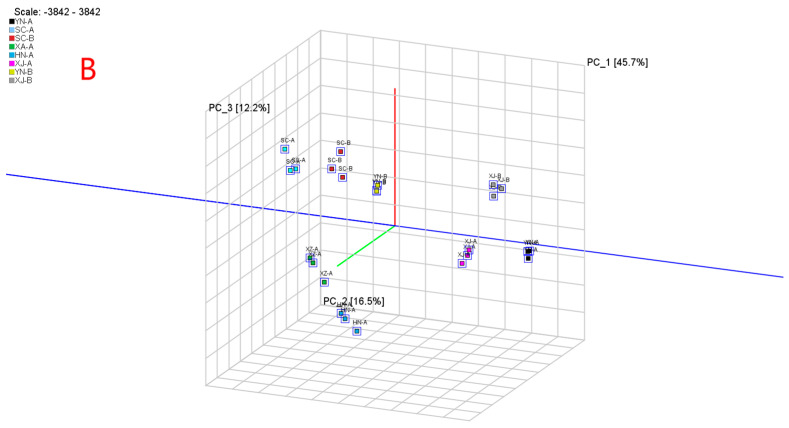
Multivariate statistical separation and model robustness. (**A**) PCA plot of PC1 vs. PC2; (**B**) 3D PCA plot of PC1, PC2, and PC3.

As shown in Figure 7A, the first two principal components explained 62% of the total variance, with PC1 accounting for 46% and PC2 for 16%, effectively capturing the major variation and enabling two-dimensional visualization. The score plot revealed distinct clustering patterns, with XJ-B located in the first quadrant; SC-A, SC-B, and YN-B in the second quadrant; XZ-A and HN-A in the third quadrant; and XJ-A together with YN-A in the fourth quadrant. To reach a cumulative explained variance of ≥70%, PC3 was included, contributing 12.2% and bringing the cumulative variance of the first three components to 74.4%. The 3D PCA plot not only confirmed the consistency of technical replicates but also provided a more intuitive visualization of the overall differences among samples, demonstrating the representativeness and explanatory power of the model.

As shown in Figure 7B, the 3D PCA plot simultaneously illustrated the VOC composition differences in the eight samples along three orthogonal axes, with a cumulative explained variance of 74.4%. The tight clustering of triplicate measurements for each sample confirmed the high reproducibility of the analysis. For example, SC-A samples were clustered within the range of negative PC1 (≈−3500), positive PC2 (≈+2500), and positive PC3 (≈+1500). In contrast, XJ-B samples were positioned at positive PC1 (≈+2500), slightly positive PC2 (≈+500), and positive PC3 (≈+1200), with the three replicates almost completely overlapping.

#### 3.4.2. OPLS-DA and Permutation Test

To further elucidate the differences among safflower samples from different regions, an OPLS-DA model was constructed based on VOC data. Compared with PCA, OPLS-DA, as a supervised discriminant method, can more effectively extract class-related information and enhance group separation, and it has been widely applied in the quality evaluation and origin authentication of fruits and vegetables [22,44]. In model evaluation, R^2^X and R^2^Y are used to assess the goodness of fit, whereas Q^2^ reflects the predictive ability. Values closer to 1 indicate stronger explanatory and predictive power, and a Q^2^ greater than 0.5 is generally considered to represent acceptable predictive performance [45].

**Figure 8:**
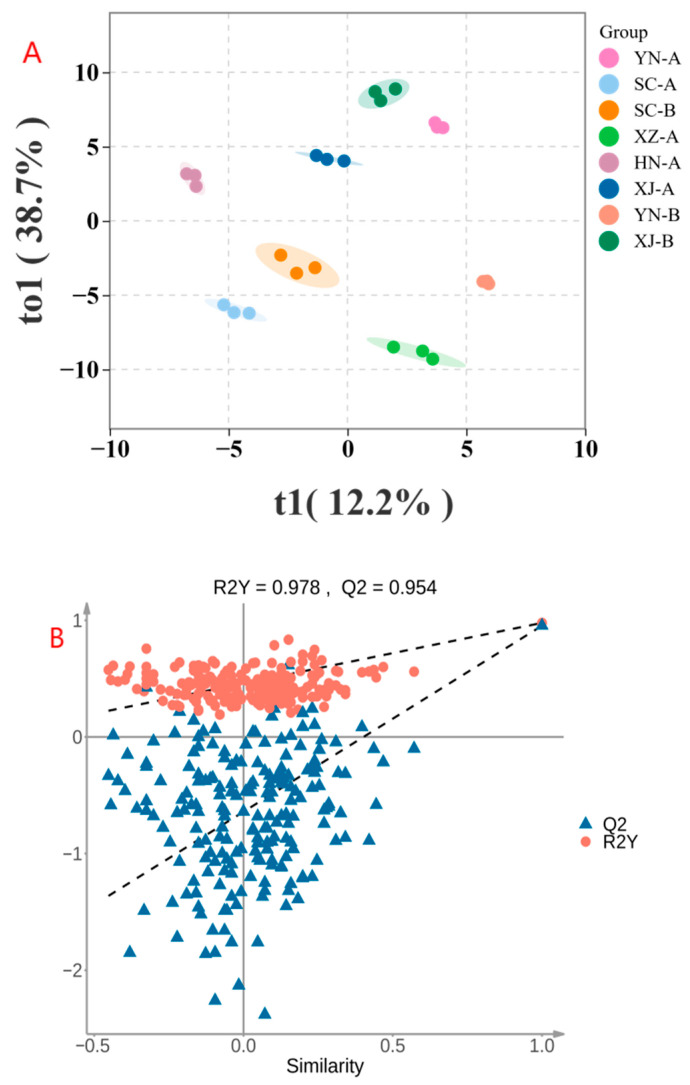
(**A**) OPLS-DA score plot; (**B**) OPLS-DA permutation test. The OPLS-DA model based on peak intensity data shows sample distribution in the two-dimensional space of t1 (12.2%, predictive component) and to1 (38.7%, orthogonal component). Samples from different regions are clearly separated along the t1 axis, and replicates cluster tightly, indicating reliable discrimination. In the permutation test, the original model exhibited markedly higher R^2^Y and Q^2^ values than all permuted models, with a negative Q^2^ intercept (R^2^Y = 0.978, Q^2^ = 0.954), confirming that the model is robust and not overfitted.

The model results indicated that safflower samples from different regions were clearly separated in the score plot, while the three replicates within each group clustered tightly, demonstrating stable differences and high reproducibility among samples (Figure 8A). Notably, XJ-B and SC-A showed the most pronounced separation, suggesting the greatest divergence in VOC composition, whereas YN-A and XJ-A were positioned more closely, reflecting their overall similarity in aroma profiles. The model achieved an R^2^Y of 0.978 and a Q^2^ of 0.954, both values approaching 1, indicating excellent goodness of fit and predictive ability.

To validate the robustness of the model, 200 permutation tests were performed [46]. The results showed that the original model exhibited markedly higher R^2^Y and Q^2^ values than all permuted models, and the Q^2^ regression line had a negative intercept when crossing the *x*-axis [47], indicating that the model was not overfitted and was highly reliable (Figure 8B).

In summary, the OPLS-DA model not only achieved effective discrimination of safflower samples from different regions but also revealed the extent of differences among key samples, which was highly consistent with the PCA and Euclidean distance analyses. These findings further demonstrate that the VOC composition of safflower exhibits significant regional characteristics, providing a solid statistical basis for subsequent biomarker selection and origin traceability studies.

### 3.5. Characteristic Volatile Compounds (VIP) and Sensory Attribution

**Figure 9:**
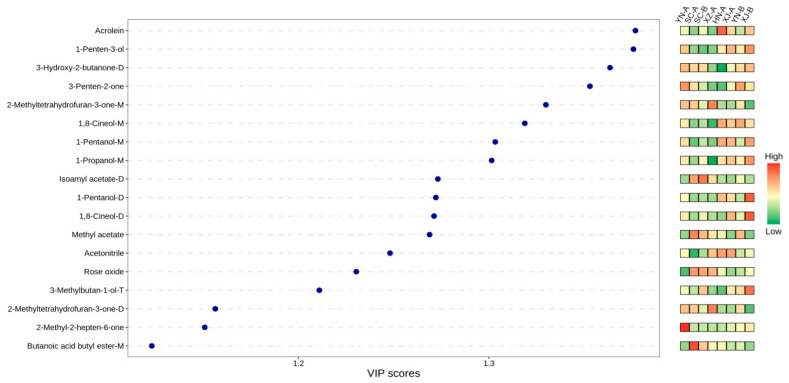
OPLS-DA variable importance in projection (VIP) bar chart. The top 18 discriminant volatile compounds are shown in descending order of VIP values, with VIP ≥ 1.

In this study, pairwise OPLS-DA comparisons of safflower volatiles from eight production regions were performed based on GC–IMS data. In these analyses, compounds with VIP > 1 are generally regarded as characteristic markers [48,49]. Therefore, a threshold of VIP ≥ 1 was applied to screen potentialregion-discriminating volatiles. The results showed that the 18 VOCs with the highest average VIP values exhibited strong discriminatory power across regions, indicating their consistent contribution to geographical differentiation. OPLS-DA variable importance in projection (VIP) analysis (Figure 9) ultimately identified these 18 compounds (VIP ≥ 1) as the key contributors to regional variation. Among them, aldehydes (e.g., acrolein and [please confirm: 1-penten-3-one or 1-penten-3-ol]), esters (e.g., methyl acetate and ethyl isovalerate), and furans (e.g., 2-methyltetrahydrofuran-3-one) were particularly important for distinguishing geographical origins. Notably, SC-A samples contained higher levels of esters and terpenes, whereas XJ-B samples were enriched in aldehydes and alcohols, highlighting pronounced differences in their aroma profiles.

**Figure 10:**
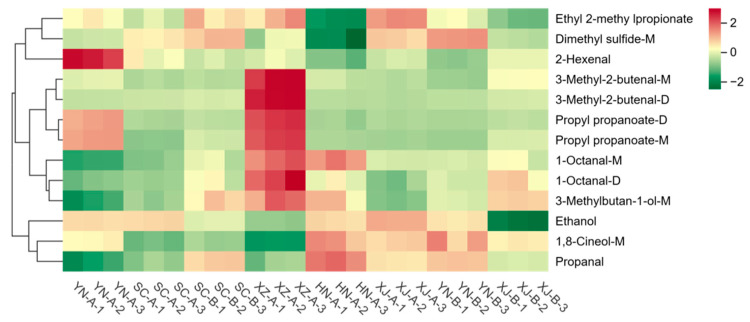
Hierarchical clustering heatmap of the identified volatiles from eight safflower samples. The *x*-axis denotes sample labels, and the *y*-axis lists volatile compounds. Green reflects lower relative levels, whereas red corresponds to higher abundance.

HCA further confirmed these results (Figure 10). Based on compounds with VIP ≥ 1 and *p* < 0.05, a total of 13 characteristic volatiles were identified, and the samples from different regions exhibited clear clustering patterns. The heatmap revealed elevated levels of several esters and terpenes in SC-A (red blocks), corresponding to floral and fruity attributes. In contrast, XJ-B showed markedly higher levels of acrolein, 1-penten-3-ol, and 3-methyl-1-butanol, resulting in a distinctive aroma profile characterized by green, pungent, and fermented notes. Samples from Tengchong, Dali, Jiangyou, and Jianyang exhibited only minor differences, mainly involving slight variations in certain aldehydes or esters. Rikaze and Xinxiang showed intermediate profiles, with the levels of some compounds similar to those in SC-A or XJ-B.

Similar regional discrimination patterns have also been reported in GC–IMS studies of tea and fruit teas [47,50], where aldehydes and esters were likewise identified as key discriminant compounds for origin authentication. In addition, studies on coffee and honey have demonstrated that GC–IMS can sensitively detect origin-related VOC differences, highlighting its broad applicability in the authentication of complex natural products [19]. Compared with these studies, the key role of furan compounds in the regional discrimination of safflower is distinctive and complements findings from investigations of spices such as turmeric and ginseng [32,33], further underscoring the unique characteristics of safflower in its aroma profile.

### 3.6. ROAV Analysis

The OPLS-DA results indicated that several compounds exhibited strong discriminatory power among safflower samples (VIP > 1), including 1,8-cineole, 2-methyltetrahydrofuran-3-one, 3-penten-2-one, and 3-methyl-1-butanol [22,29]. However, since VIP values alone do not reflect the actual contribution of these compounds to aroma perception, relative odor activity values (ROAVs) were further calculated for 16 volatiles. Table 3 summarizes representative compounds with their odor descriptions, thresholds, and ROAV ranges, directly linking chemical composition with sensory relevance and supporting the identification of regional aroma markers.

In addition to the core components, 2-methyltetrahydrofuran-3-one (ROAV > 1) contributed in all samples, imparting roasted and nutty notes. Isoamyl acetate and butyl butyrate were present at higher levels in SC-A and SC-B, providing fruity and milky aromas that enhanced the overall aroma complexity. Notable differences were observed among the samples: the ROAV of 1,8-cineole in XJ-B (68.76) was markedly higher than in SC-A (17.39), which may be related to differences in LOX/HPL enzyme activity [51,52]. In contrast, the ROAV of acrolein in SC-A was relatively low (0.86), likely resulting from enhanced lipid peroxidation under strong solar radiation and low-humidity conditions. Similarly, ketones such as 2-methyl-2-hepten-6-one were lower in SC-A than in XJ-B, consistent with their formation via microbial thermal oxidation and Maillard reactions [53]. In terms of esters, butyl butyrate and 2-methyl-1-propyl acetate were present at lower levels in XJ-B than in SC-A. Notably, the ROAV of 2-methyl-1-propyl acetate in XJ-B was <1, indicating only a modifying role, suggesting that fruity–sweet ester notes are not characteristic of this sample. This change may be associated with limited catalysis by alcohol acyltransferase (AAT), enhanced ester hydrolysis, and accelerated volatilization under high-temperature, low-humidity conditions [22].

In terms of overall aroma characteristics, YN-A exhibited the highest ROAV, exhibiting a complex floral–fruity–fresh profile. SC-A and SC-B were dominated by fruity notes, mainly driven by butyl butyrate and isoamyl acetate. XZ-A showed generally lower ROAVs, which resulted in a relatively simple aroma profile. Comparative analysis further revealed that 3-methyl-1-butanol was more abundant in XJ-B (ROAV ≈ 8), contributing fermented and alcoholic nuances. Finally, 2-methyltetrahydrofuran-3-one showed ROAV > 1 across all samples and was likely derived from lipid oxidation and Maillard reactions [54,55], imparting roasted and nutty notes to the overall aroma.

## 4. Conclusions

This study employed GC–IMS combined with heatmap analysis, PCA, OPLS-DA, and ROAV to systematically profile the volatile organic compounds (VOCs) in safflower samples from eight major producing regions in China. In total, 140 VOCs were detected, of which 108 were identified, predominantly belonging to aldehydes, esters, and alcohols. The results consistently demonstrated significant regional differences in the volatile composition of safflower. Several compounds exhibited region-specific enrichment and could serve as potential markers for origin discrimination, including propyl acetate, isoamyl acetate, and butyl butyrate in SC-A; hexanal and nonanal in SC-B; dimethyl disulfide and 6-methyl-5-hepten-2-one in YN-A; 3-methyl-3-buten-1-ol and ethyl acetate in YN-B; dimethyl sulfide in XJ-A; hexanal, nonanal, 3-methyl-1-butanol, and 1,8-cineole in XJ-B; 3-methyl-2-butenal and octanal in XZ-A; and acrolein and pentanal in HN-A. In contrast, 2-methyltetrahydrofuran-3-one (ROAV > 1) contributed to aroma complexity but lacked discriminative value.

Overall, safflower VOC profiles show clear geographic differentiation. GC–IMS coupled with chemometric analyses provides robust origin discrimination, objective quality evaluation, and marker discovery. These findings are consistent with GC–IMS studies on tea, chrysanthemum, and saffron, and they highlight the recurring importance of aldehydes and esters in geographic traceability. In addition, the preferential occurrence of sulfur-containing volatiles and oxygenated ketones distinguishes the safflower aroma. Validation with authenticated GC–MS standards, together with sensory testing, should increase confidence in these markers and clarify their links to consumer perception. The same workflow is applicable to safflower-derived products (e.g., safflower tea, extracts, and oil) for routine quality control; combined with sensory analysis, it enables an integrated chemical–sensory assessment useful for food quality monitoring and flavor research.

## Figures and Tables

**Table 1:** Geographical location and climatic characteristics of eight safflower (*Carthamus tinctorius* L.) sampling sites in China.

Code	Location	Latitude	Longitude	Elevation	Climate Type	Mean Annual Precip.
HN-A	Henan (Xinxiang), China	35.3086° N	114.0511° E	73 m	Warm-temperate monsoon climate	Annual precipitation ranges from approximately 600 to 700 mm, with the majority occurring during the summer months.
SC-A	Sichuan (Jianyang), China	31.7774° N	104.7421° E	400–580 m	Humid subtropical climate	Approximately 900–1100 mm.
SC-B	Sichuan (Jiangyou), China	30.3931° N	104.5532° E	800–1500 m	Humid subtropical climate	Approximately 1000–1200 mm.
XZ-A	Rikaze (Tibet Autonomous Region), China	28.3683° N	87.7634° E	3836 m	Alpine plateau climate	Approximately 400 mm, characterized by arid and low-rainfall conditions.
XJ-A	Xinjiang (Yili), China	43.9052° N	81.2747° E	1600–1900 m	Temperate continental climate with oasis irrigation	Approximately 400–500 mm, representing a relatively humid area within Xinjiang.
XJ-B	Xinjiang (Tacheng), China	46.7510° N	82.9838° E	1100–5000 m	Arid continental climate	Approximately 200–250 mm, extremely arid.
YN-A	Yunnan (Tengchong), China	25.017° N	98.483° E	1640 m	Mild subtropical highland climate	Approximately 1500 mm, with a long rainy season.
YN-B	Yunnan (Dali), China	25.6065° N	100.2676° E	1970 m	Subtropical plateau monsoon climate	Approximately 1050 mm.

**Table 2:** Euclidean distance and similarity analysis.

Full Distance	HN-A	SC-A	SC-B	XJ-A	XJ-B	XZ-A	YN-A	YN-B
HN-A	0	17.71778	15.83031	15.32197	20.15048	15.10411	18.67716	15.35231
SC-A	17.71778	0	11.14107	19.78153	23.81304	14.76061	22.18853	12.94494
SC-B	15.83031	11.14107	0	15.0435	18.69994	13.83421	19.0351	8.945528
XJ-A	15.32197	19.78153	15.0435	0	15.79017	17.19138	12.04877	12.04061
XJ-B	20.15048	23.81304	18.69994	15.79017	0	23.18586	15.69517	18.25957
XZ-A	15.10411	14.76061	13.83421	17.19138	23.18586	0	20.74933	14.09947
YN-A	18.67716	22.18853	19.0351	12.04877	15.69517	20.74933	0	16.9434
YN-B	15.35231	12.94494	8.945528	12.04061	18.25957	14.09947	16.9434	0

Note: Each cell represents the Euclidean distance between sample pairs (calculated based on the peak intensity matrix after log_1_p transformation and z-score normalization). A smaller distance indicates higher similarity. The median value of three parallel replicates was used for comparison.

**Table 3:** Representative volatile organic compounds (VOCs) in safflower (*Carthamus tinctorius* L.) from eight production regions, with relative abundance and ROAVs.

No.	Compound	CAS	RI	Odor Description	Odor Threshold (mg/kg)	ROAV Range (Across Samples)	Key Sample Enrichment
1	Hexanal	C66251	1090.3	Green, grassy, fatty	4.50	15–42	SC-B, XJ-B
2	Nonanal	C124196	1399.1	Waxy, citrus, oily	1	12–35	SC-B, XJ-B
3	3-Methylbutanoic acid	C503742	1669.4	Fruity, sweet	30	18–40	YN-B
4	Butanoic acid	C107926	1625.5	Fruity, fermented	42	4–10	XJ-B
5	2-Methyl-1-propyl acetate	C110190	1021.0	Fruity, raw pear and raspberrie	0.01	4.5–10	SC-B
6	Isoamyl acetate	C123922	1122.5	Fruity, banana, sweet	17	7–20	SC-A, SC-B
7	Butanoic acid butyl ester	C109217	1219.2	Fruity, pineapple, sweet	4.80	8–19	SC-A
8	Rose oxide	C16409431	1341.9	Floral, rose-like	0.32	57–100	YN-A, SC-B
9	1,8-Cineole	C470826	1204.7	Camphor, herbal, cooling	1.20	17–100	XJ-B
10	3-Penten-2-one	C625332	1133.0	Fruity, spicy	0.94	16–87	YN-A
11	Acetic acid	C64197	1464.3	Sulfurous, onion-like	0.05	5–20	YN-A
12	2-Furaldehyde	C98011	1458.4	sweet, woody, almond, bready	0.02	3–12	XJ-A
13	Acrolein	C107028	870.3	Pungent, acrid	105	0.8–3.9	HN-A
14	2-Methyl-2-hepten-6-one	C110930	1355.9	Citrus, fruity	50	0.3–9.3	YM-A
15	2-Methyltetrahydrofuran-3-one	C3188009	1355.9	Roasted, nutty, buttery	2	7.4–29	All samples

Note: Full list of 140 VOCs is provided in Supplementary Table S1. ROAVs > 1 indicate key aroma contributors; 0.1–1, modifiers; <0.1, negligible impact. This table presents 15 representative compounds, while the complete list is provided in Supplementary Table S1.

## Data Availability

The original contributions presented in this study are included in the article/Supplementary Material, and further inquiries can be directed to the corresponding author.

## Supplementary Materials

The following supporting information can be downloaded at: https://www.mdpi.com/article/10.3390/foods14193381/s1, Table S1: Comprehensive profile of volatile compounds identified in safflower.
