# Supplementary material for: Volatile Fingerprinting and Regional Differentiation of Safflower (Carthamus tinctorius L.) Using GC–IMS Combined with OPLS-DA
Source: Foods. 2025 Sep 29;14(19):3381. doi: 10.3390/foods14193381 (PMC12523545; doi:10.3390/foods14193381)
Supplement: Supplementary file 1 [file foods-14-03381-s001.zip › foods-3854549-supplementary.pdf]

Table S1. Comprehensive profile of volatile compounds identified in safflower.

No.	Compound	CAS#	Formula	MW （Da）	RI	Rt (s)	Dt（RIPrel.）	Flavour description
1	Pentanoic acid	C109524	C5H10O2	102.1	1736.3	1914.593	1.23988	fatty, decay, slightly sour, slightly sweet）
2	3-Methyl butyric acid	C503742	C5H10O2	102.1	1669.4	1559.872	1.22747	sour, foot sweat, cheese
3	Butanoic acid	C107926	C4H8O2	88.1	1625.5	1364.202	1.17237	strong acetic acid, cheese, butter, fruity
4	2-Methyl propanoic acid	C79312	C4H8O2	88.1	1572.3	1191.77	1.15567	yogurt, rancid cream
5	Propanoic acid	C79094	C3H6O2	74.1	1537.3	1090.523	1.10733	yogurt, vinegar
6	Acetic acid	C64197	C2H4O2	60.1	1464.3	905.899	1.06128	spicy
7	2-Furaldehyde	C98011	C5H4O2	96.1	1458.4	892.543	1.33522	sweet, woody, almond, bready
8	1-Nonanal	C124196	C9H18O	142.2	1399.1	767.652	1.47975	rose, citrus, strong oily
9	1-Hexanol	C111273	C6H14O	102.2	1372.2	720.287	1.33336	fresh, fruity, wine, sweet, green
10	(Z)-2-Penten-1-ol	C1576950	C5H10O	86.1	1335.5	661.408	0.9477	green, plastic, rubber
11	2-Methyl-2-hepten-6-one	C110930	C8H14O	126.2	1355.9	693.395	1.18341	citrus, fruity, mouldy, ketone
12	(E)-2-Heptenal	C18829555	C7H12O	112.2	1338.8	666.379	1.25876	spicy, green vegetables, fresh, fatty
13	1-Hydroxy-2-propanone	C116096	C3H6O2	74.1	1321.0	639.362	1.0591	pungent, caramel, fresh
14	3-Hydroxy-2-butanone	C513860	C4H8O2	88.1	1297.6	605.496	1.06035	butter, cream
15	1-Octanal	C124130	C8H16O	128.2	1306.1	617.673	1.41195	aldehyde, waxy, citrus, orange, fruity, fatty
16	2-Methyltetrahydrofuran-3-one	C3188009	C5H8O2	100.1	1273.7	565.11	1.42693	sweet, bread, buttery, nutty
17	1-Pentanol	C71410	C5H12O	88.1	1260.7	543.325	1.25891	balsamic
18	3-Methyl-3-buten-1-ol	C763326	C5H10O	86.1	1259.3	541.15	1.17251	sweet, fruity
19	(Z)-4-Heptenal	C6728310	C7H12O	112.2	1249.0	524.635	1.14718	grass, oil
20	(E)-2-Hexenal	C6728263	C6H10O	98.1	1222.8	484.865	1.18468	green, banana, fat
21	3-Methylbutan-1-ol	C123513	C5H12O	88.1	1208.4	464.282	1.24449	whiskey, banana, fruity
22	3-Methyl-2-butenal	C107868	C5H8O	84.1	1203.2	457.063	1.0903	fruity

No.	Compound	CAS#	Formula	MW （Da）	RI	Rt (s)	Dt（RIPrel.）	Flavour description
23	Limonene	C138863	C10H16	136.2	1192.2	442.28	1.22247	lemon, sweet, orange, pine oil
24	Heptanal	C111717	C7H14O	114.2	1185.2	432.998	1.32893	fresh, aldehyde, fatty, green herbs, wine, fruity
25	1-Penten-3-ol	C616251	C5H10O	86.1	1162.5	399.146	0.95101	ethereal, green, tropical fruity
26	(Z)-2-Methylpent-2-enal	C623369	C6H10O	98.1	1152.4	384.608	1.16339	aldehydes, soil, garlic, ripe cherries, fruity
27	1-Butanol	C71363	C4H10O	74.1	1147.3	377.401	1.18921	wine
28	Butyl propanoate	C590012	C7H14O2	130.2	1141.3	369.126	1.28511	earthy, sweet， rose
29	(E)-2-Pentenal	C1576870	C5H8O	84.1	1135.6	361.385	1.3601	potato, peas
30	3-Penten-2-one	C625332	C5H8O	84.1	1133.0	357.915	1.0761	Fruity, turns into spicy during storage
31	2-Pentanol	C6032297	C5H12O	88.1	1120.8	342.166	1.21011	Fusel Oil, Green
32	Isoamyl acetate	C123922	C7H14O2	130.2	1122.5	344.302	1.30601	sweet, banana, fruity
33	Hexanal	C66251	C6H12O	100.2	1090.3	305.596	1.28511	fresh, green, fat, fruity
34	Butyl acetate	C123864	C6H12O2	116.2	1079.0	294.652	1.24208	fruity
35	Ethyl 3-methylbutanoate	C108645	C7H14O2	130.2	1071.0	287.383	1.26995	apple, banana, sour and sweet
36	Dimethyl disulfide	C624920	C2H6S2	94.2	1075.1	291.081	1.13692	sulfurous , cabbage, onion
37	(E)-2-Butenal	C123739	C4H6O	70.1	1055.9	274.271	1.04794	null
38	Propyl propanoate	C106365	C6H12O2	116.2	1045.2	265.345	1.21614	pineapple
39	1-Penten-3-one	C1629589	C5H8O	84.1	1033.6	256.003	1.07941	strong pungent odors
40	Methyl 3-methylbutanoate	C556241	C6H12O2	116.2	1027.0	250.847	1.52258	strong apple，pineapple
41	2-Methylbutanoic acid methyl ester	C868575	C6H12O2	116.2	1017.6	243.644	1.53454	apple
42	Acetonitrile	C75058	C2H3N	41.1	1021.0	246.246	1.02863	floral
43	2-Pentanone	C107879	C5H10O	86.1	992.1	225.164	1.37326	acetone, fresh, sweet fruity, wine
44	n-Pentanal	C110623	C5H10O	86.1	993.2	225.906	1.42956	green grassy, faint banana, pungent

No.	Compound	CAS#	Formula	MW（Da）	RI	Rt (s)	Dt（RIPrel.）	Flavour description
45	Propyl acetate	C109604	C5H10O2	102.1	964.9	208.53	1.47575	fruity, pear
46	Ethanol	C64175	C2H6O	46.1	944.9	197.256	1.12717	aromaticity
47	2-Butanone	C78933	C4H8O	72.1	916.9	182.6	1.24598	fruity , camphor
48	3-Methyl butanal	C590863	C5H10O	86.1	927.9	188.237	1.40178	chocolate, fat
49	Methanol	C67561	CH4O	32.0	908.4	178.316	1.0319	alcohol, pungent
50	Butanal	C123728	C4H8O	72.1	894.2	171.443	1.28453	pungent, fruity, green leaf
51	Acetic acid ethyl ester	C141786	C4H8O2	88.1	902.0	175.197	1.34081	fresh, fruity, sweet, grassy
52	Acrolein	C107028	C3H4O	56.1	870.3	160.469	1.06321	strong pungent
53	Methyl acetate	C79209	C3H6O2	74.1	857.4	154.842	1.1961	Ester, Green
54	Acetone	C67641	C3H6O	58.1	845.2	149.712	1.12079	fresh, apple, pear
55	Propanal	C123386	C3H6O	58.1	822.5	140.611	1.14663	pungent, green grassy
56	2-Methyl propanal	C78842	C4H8O	72.1	836.7	146.237	1.2847	banana, melon , slightly nutty
57	Dimethyl sulfide-M	C75183	C2H6S	62.1	803.3	133.329	0.95837	cabbage, sulfur, gasoline
58	Acetaldehyde	C75070	C2H4O	44.1	775.3	123.4	0.98125	green, slight fruity
59	Benzaldehyde	C100527	C7H6O	106.1	1492.3	972.687	1.15319	bitter almond, cherry, nutty
60	Ethyl 2-methylpropionate	C97621	C6H12O2	116.2	970.7	211.869	1.57274	sweet, fruity, alcoholic, rummy
61	Butanoic acid butyl ester	C109217	C8H16O2	144.2	1219.2	479.7	1.34256	fruity, banana, pineapple, green cherry , tropical fruit mature and juicy fruity aroma
62	2-Methyl-1-propanol	C78831	C4H10O	74.1	1099.0	315.649	1.17125	fresh, alcoholic, leather
63	1-Propanol	C71238	C3H8O	60.1	1046.3	266.237	1.1105	alcohol, pungent
64	1,8-Cineol	C470826	C10H18O	154.3	1204.7	459.178	1.30467	camphor, refreshing herbal
65	2-Methyl-1-propyl acetate	C110190	C6H12O2	116.2	1021.0	246.255	1.61616	fruity, raw pear and raspberrie
66	Unknown	unidentified	*	0	1779.7	2186.33	1.12687	null

No.	Compound	CAS#	Formula	MW （Da）	RI	Rt (s)	Dt（RIPrel.）	Flavour description
67	Unknown	97	*	0	1574.4	1198.002	1.08886	null
68	Unknown	unidentified	*	0	1530.2	1070.882	1.1463	null
69	Unknown	unidentified	*	0	1533.3	1079.524	1.36356	null
70	Unknown	unidentified	*	0	1498.6	988.39	1.22777	null
71	Unknown	unidentified	*	0	1553.2	1135.304	1.27028	null
72	Unknown	unidentified	*	0	1383.4	739.264	1.09658	null
73	Unknown	unidentified	*	0	1345.6	677.101	0.95058	null
74	Unknown	unidentified	*	0	1357.5	696.059	1.10933	null
75	Unknown	unidentified	*	0	1355.4	692.634	1.4446	null
76	Unknown	unidentified	*	0	1231.0	496.998	1.0813	null
77	Unknown	unidentified	*	0	1230.8	496.661	1.31846	null
78	Unknown	unidentified	*	0	1175.7	419.247	1.27142	null
79	Unknown	unidentified	*	0	1175.1	418.216	1.68261	null
80	Unknown	unidentified	*	0	1160.6	396.354	1.48796	null
81	Unknown	unidentified	*	0	1159.7	395.019	1.58631	null
82	Unknown	unidentified	*	0	1153.4	385.943	1.77687	null
83	Unknown	unidentified	*	0	1153.9	386.744	1.32199	null
84	Unknown	unidentified	*	0	1115.1	334.959	1.11053	null
85	Unknown	unidentified	*	0	1104.5	322.146	1.09577	null
86	Unknown	unidentified	*	0	1105.2	322.947	1.35272	null
87	Unknown	unidentified	*	0	1044.3	264.636	0.94519	null
88	Unknown	unidentified	*	0	1005.1	234.433	1.06879	null
89	Unknown	unidentified	*	0	969.3	211.073	1.71186	null

No.	Compound	CAS#	Formula	MW（Da）	RI	Rt (s)	Dt（RIPrel.）	Flavour description
90	Unknown	unidentified	*	0	961.4	206.5	1.18994	null
91	Unknown	unidentified	*	0	946.1	197.932	1.38272	null
92	Unknown	unidentified	*	0	1118.9	339.787	1.09088	null
93	Unknown	unidentified	*	0	1005.0	234.325	1.31978	null
94	Unknown	unidentified	*	0	1107.4	325.632	1.14181	null
95	Unknown	unidentified	*	0	1111.6	330.67	1.20081	null
96	Unknown	unidentified	*	0	1110.8	329.732	1.42229	null
97	gamma-Butyrolactone	C96480	C4H6O2	86.1	1615.7	1330.687	1.09941	cream, fat, caramel
98	Isophorone	C78591	C9H14O	138.2	1600.2	1279.12	1.25775	camphor
99	Decanal	C112312	C10H20O	156.3	1483.3	950.599	1.53709	sweet, waxy, floral, citrus, aldehyde, fatty
100	Rose oxide	C16409431	C10H18O	154.3	1341.9	671.263	1.35545	green, rose, floral
101	Butyl 3-methylbutanoate	C109193	C9H18O2	158.2	1296.6	604.154	1.39459	bananas, blue cheese
102	gamma-Terpinene	C99854	C10H16	136.2	1246.3	520.316	1.21596	oil, wood, terpenes, lemon, lime, herbs
103	Unknown	unidentified	*	0	1257.8	538.614	1.21484	null
104	2-Hexenal	C505577	C6H10O	98.1	1204.9	459.467	1.18197	sweet almonds, fruity, green leaves, apples, plums, vegetables
105	Cyclopentanone	C120923	C5H8O	84.1	1183.8	431.179	1.10562	pleasant
106	Allyl sulfide	C592881	C6H10S	114.2	1132.0	356.55	1.11062	garlic
107	4-Methyl-2-pentanone	C108101	C6H12O	100.2	1012.7	239.972	1.17822	ketone
108	2-Ethyl furan	C3208160	C6H8O	96.1	964.9	208.512	1.04872	bean, bread, malt, caramel

Note: Compounds labeled as “Unknown” were detected by GC–IMS but could not be precisely identified due to lack of matching standards or database references.GC–IMS monomer/dimer ion species (M/D) were not treated as distinct compounds.
